# Immunohistological Examination of AKT Isoforms in the Brain: Cell-Type Specificity That May Underlie AKT’s Role in Complex Brain Disorders and Neurological Disease

**DOI:** 10.1093/texcom/tgab036

**Published:** 2021-05-28

**Authors:** Josien Levenga, Helen Wong, Ryan Milstead, Lauren LaPlante, Charles A Hoeffer

**Affiliations:** 1 Institute for Behavioral Genetics, University of Colorado Boulder, Boulder, CO 80303, USA; 2 Department of Integrative Physiology, University of Colorado Boulder, Boulder, CO 80303, USA; 3 Linda Crnic Institute, Anschutz Medical Campus, Aurora, CO 80045, USA

**Keywords:** amygdala, astrocytes, hippocampus, interneurons, microglia

## Abstract

Protein kinase B (PKB/AKT) is a central kinase involved in many neurobiological processes. AKT is expressed in the brain as three isoforms, AKT1, AKT2, and AKT3. Previous studies suggest isoform-specific roles in neural function, but very few studies have examined AKT isoform expression at the cellular level. In this study, we use a combination of histology, immunostaining, and genetics to characterize cell-type-specific expression of AKT isoforms in human and mouse brains. In mice, we find that AKT1 is the most broadly expressed isoform, with expression in excitatory neurons and the sole detectable AKT isoform in gamma-aminobutyric acid ergic interneurons and microglia. By contrast, we find that AKT2 is the sole isoform expressed in astroglia and is not detected in other neural cell types. We find that AKT3 is expressed in excitatory neurons with AKT1 but shows greater expression levels in dendritic compartments than AKT1. We extend our analysis to human brain tissues and find similar results. Using genetic deletion approaches, we also find that the cellular determinants restricting AKT isoform expression to specific cell types remain intact under *Akt* deficiency conditions. Because AKT signaling is linked to numerous neurological disorders, a greater understanding of cell-specific isoform expression could improve treatment strategies involving AKT.

## Introduction

Neurological diseases and disorders have complex etiologies with genetics and different brain cell types, which are known to play critical roles in both their manifestation and treatment (Sullivan et al. 2003; Schumacher et al. 2011; Dienel and Lewis 2019; Musci et al. 2019; Verkhratsky et al. 2019). The effectiveness of available therapies is still quite limited, and many patients remain untreated (Gould et al. 2007; Duman and Voleti 2012). Improved understanding of critical molecular signaling pathways underlying neurological dysfunction is necessary to enhance and develop novel therapeutic targets. A diverse body of genetic and pharmacological work implicates the protein kinase B (PKB/AKT) family of serine/threonine kinases in many neurological and psychiatric disorders (Chalecka-Franaszek and Chuang 1999; De Sarno et al. 2002; Beaulieu et al. 2004; Emamian et al. 2004; Ikeda et al. 2004; Alimohamad et al. 2005; Li et al. 2007; Beaulieu et al. 2008; Tan et al. 2008; Beaulieu et al. 2009; Karege et al. 2010; Blasi et al. 2011; Karege et al. 2012; Pereira et al. 2014; Schizophrenia Working Group of the Psychiatric Genomics 2014). AKT is expressed as three isoforms termed AKT1/PKBα, AKT2/PKBβ, and AKT3/PKBγ in the brain. Although each isoform shows significant homology with one another and across species (Manning and Cantley 2007), they exhibit different effects on behavior and neural function (Balu et al. 2012; Leibrock et al. 2013; Chang et al. 2016; Bergeron et al. 2017; Howell et al. 2017; Levenga et al. 2017; Wang et al. 2017). Recently, we demonstrated that AKT isoforms participate differently in synaptic plasticity and protein synthesis regulation (Levenga et al. 2017). We also showed that AKT isoforms exhibit some cell-type expression pattern differences in the hippocampus (Levenga et al. 2017).

The brain comprises many cell types, including diverse neuronal populations (Masland 2004; Lodato and Arlotta 2015). In addition to excitatory neurons, which use glutamate for neurotransmission, interneurons exert predominantly inhibitory signaling on other neurons through gamma-aminobutyric acid (GABA)ergic transmission (DeFelipe et al. 2013; Pelkey et al. 2017). Interneurons can be identified by the expression of glutamate decarboxylase-67 (GAD67) (Stone et al. 1999) and are composed of at least five major subclasses that can be distinguished with molecular markers, such as parvalbumin (PV), somatostatin (SST), and calretinin (CAL) (Mayer et al. 2018). The brain also contains nonneuronal cells called glia (Masland 2004). Glia serve several essential functions, including homeostatic maintenance, modulation of neurotransmission, myelin formation, and responses to injury and disease (Simard and Nedergaard 2004; Barres 2008; Schummers et al. 2008; Perea et al. 2009; Santello et al. 2019). Astrocytes are the most abundant glial cells in the brain. In a healthy central nervous system (CNS), they participate in neurodevelopment, blood flow regulation, ion and neurotransmitter balance, blood–brain barrier (BBB) formation, and synaptic function (Simard and Nedergaard 2004; Barres 2008; Perea et al. 2009; Santello et al. 2019). Astrocytes can be identified by the expression of glial fibrillary acidic protein (GFAP) and aldehyde dehydrogenase 1 family member L1 (ALDH1L1) (Serrano-Pozo et al. 2013; Hol and Pekny 2015). Another type of glia is microglia, which are the resident immune (macrophage) cells of the brain and play vital roles in the synaptic maintenance and removal of damaged cells and tissues (von Bernhardi et al. 2016). Microglia can be identified by the expression of ionized calcium-binding adapter molecule 1 (IBA1), a protein involved in microglial activation (Ahmed et al. 2007). Different brain cell types are known to contribute to the expression of human neurological diseases; therefore, improved understanding of cell-specific signaling cascades may provide the means to more effectively diagnose and treat neurological disorders (Sullivan et al. 2003; Schumacher et al. 2011; Dienel and Lewis 2019; Musci et al. 2019; Verkhratsky et al. 2019).

We previously provided some evidence for cell-type-specific expression for AKT isoforms in the hippocampal area CA1 of the mouse brain (Levenga et al. 2017). This study expands this characterization to other areas of the hippocampus and additional brain regions and defines AKT isoform specificity in more brain cell types. Importantly, we examine AKT isoform expression in fully developed adult human brain tissue and establish for the first time that AKT isoform cell-type expression specificity observed in the mouse brain may also be represented in humans. Finally, we show that restricted AKT protein expression is maintained in a cell-type-specific fashion even under conditions of AKT isoform deficiency. These data explain the expression of multiple AKT isoforms in the brain. These findings may improve our understanding of the PI3K/AKT/mTOR pathway’s role in human neuropsychiatric disorders and neurodegenerative diseases.

## Materials and Methods

### Mice

Combinations of conditional and global *Akt* isoform knockout (KO) were generated in the C57BL/6 background. *Akt* isoform KO mice were produced as previously described (Levenga et al. 2017). For conditional *Akt1* removal from the excitatory neurons in the forebrain, we bred *Akt1^fl/fl^* (Jackson Laboratory) with *Camk2a-*Cre (T29-1) mice. For conditional removal of *Akt1* or *Akt2* from the whole brain, we bred *Akt1^fl/fl^* and *Akt2^fl/fl^* (Jackson Laboratory) with nestin (*Nes*)-Cre (Jackson Laboratory) mice. For the removal of more than one *Akt* isoform, floxed *Akt* isoform mice were crossed together or into the *Akt3* KO line and used in combination with the Cre lines to create *Nes*-Cre; *Akt2^fl/fl^*; *Akt3* KO (n2F3K), *Camk2a-*Cre; *Akt1^fl/fl^*; *Akt3* KO (c1F3K), or *Nes*-Cre; *Akt1^fl/fl^*; *Akt2^fl/fl^* (n1F2F) mice. Littermates carrying the wild-type (WT) alleles or lacking the transgenes were used as controls. Genotypes were determined by PCR of ear or tail deoxyribonucleic acid (DNA) using the primer sets described in previous reports (Levenga et al. 2017) or by Jackson Laboratory. Mice were group-housed in the same facility and were maintained on a 12:12 h light:dark schedule with food and water available ad libitum. At 3–5 months old, mice of both sexes were anesthetized using a mixture of pentobarbital sodium and phenytoin sodium (Euthanasia III). When they were unresponsive to a toe pinch, mice were transcardially perfused with phosphate-buffered saline (PBS) followed by 4% paraformaldehyde (PFA). Brains were then postfixed with 4% PFA for 24 h at 4 °C, transferred to 30% sucrose in PBS for 24 h minimum at 4 °C, sectioned into 30-μm coronal slices on a cryostat (Leica), and stored free-floating at −20 °C in cryoprotectant (30% sucrose/30% ethylene glycol in phosphate buffer) until used for immunohistology. All procedures were approved by the University of Colorado Boulder Institutional Animal Care and Use Committee and conformed to the National Institutes of Health’s “Guide for the Care and Use of Laboratory Animals.”

### Mouse Brain Tissue Immunostaining

Fluorescent immunostaining was performed as described previously with minor changes (Levenga et al. 2013). Briefly, free-floating brain sections were washed with PBS-T (1X PBS containing 0.5% TritonX-100) and were submitted to heat-mediated antigen retrieval in citrate buffer (10 mM, pH 6, boiling for 10 min) if needed. Slices were blocked for 1 h at room temperature (RT) in staining buffer containing 0.05 M Tris pH 7.4, 0.9% NaCl, 0.25% gelatin, 0.5% TritonX-100, and 5% donkey serum. Slices were then incubated for 40 h at 4 °C with a combination of primary antibodies against AKT1 (1:250, Cell Signaling D9R8K), AKT2 (1:250, Cell Signaling 5B5), AKT3 (1:250, Cell Signaling E1Z3W), NeuN (1:1000, Millipore Mab377 or 1:500, Synaptic systems 266 006), CaMKIIα (1:500, Abcam ab22609), PV (1:1000, Millipore MAB1572), GAD67 (1:1500, Millipore MAB5406), SST (1:150, Novus NBP100–64650), CAL (1:500, Swant CG1), ALDH1L1 (1:300, Novus NBP2-50033), IBA1 (1:500, Synaptic Systems 234 004), or GFAP (1:1000, PhosphoSolutions 621-GFAP) diluted in staining buffer. Specificity of AKT1, AKT2, and AKT3 antibodies were confirmed previously (Levenga et al. 2017). After three washes in PBS-T, the brain slices were incubated at RT for 2 h in a combination of Cy3-conjugated antirabbit; Alexa Fluor 488-conjugated antimouse, antimouse IgG2A, antiguinea pig, antichicken, or antirat; and Alexa Fluor 647-conjugated antimouse, antimouse IgG2A, antiguinea pig, antigoat, or antichicken secondary antibodies (1:200, Jackson ImmunoResearch or Thermo Fisher Scientific) diluted in staining buffer without donkey serum and with Hoechst dye (1:1000). Following two washes in PBS-T and one wash in PBS, slices were mounted and coverslipped with Mowiol. Z-stacks through the entire thickness of the brain slices were imaged using the Nikon A1R confocal microscope with all microscope parameters held constant across slices from the same experiment. Images are representative of three independent samples for each staining.

### AKT Signal Quantification

To quantify AKT isoform levels in different cell types, a 100-μm^2^ region of interest (ROI) was created around the soma of neurons (NeuN+/Hoechst+), interneurons (PV+/Hoechst+), or astrocytes (GFAP+/Hoechst+) in the maximum intensity projection of Z-stacks from the hippocampus. AKT isoform signal was measured as the sum pixel intensity of Cy3 fluorescence in the ROI. Intensity measurements were then averaged by the cell type for each isoform and were subtracted by the average background intensity in ROIs obtained from *Akt* isoform KO slices. Three to five cells of each type per mouse were analyzed. To quantify AKT1 and AKT3 levels in hippocampal subregions, a 2000-μm^2^ ROI was created through the stratum pyramidale (SP) or stratum radiatum (SR) of area CA1 or CA3, and the sum pixel intensity of Cy3 fluorescence in the ROI was measured. Intensity measurements were then averaged by the hippocampal region for AKT1 and AKT3 and were subtracted by the average background intensity in ROIs obtained from *Akt1* or *Akt3* KO slices, respectively. Two to four slices per mouse were analyzed. Data were obtained from three to four mice of mixed sexes for each experiment and were presented as the mean ± standard error of the mean (SEM). Statistical significance was determined by two-tailed Student’s *t*-test or two-way ANOVA where appropriate using Prism (GraphPad).

### Brain Injury Procedure

Glial activation was induced by brain injury following sham surgery on 3-month-old C57BL/6 mice weighing 25–28 g. For surgery, mice were anesthetized with inhaled isoflurane and stabilized in a stereotactic frame (David Kopf Instruments) on a heated pad throughout the procedure. Using aseptic technique, the skull was exposed and a burr-hole of 0.5 mm in diameter was drilled to lower a 30-G needle into the hippocampus at Bregma coordinates: −1.94 mm anterioposterior, ±1.75 mm mediolateral, and − 2 mm dorsoventral. The needle was then retracted slowly, and the surgical wound was closed. Mice were returned to their home cages after a subcutaneous injection of buprenorphine-SR (0.05 mg/kg) and were allowed to recover. At 72 h postsurgery, the brains from these mice were fixed and sectioned for microscopy as described above.

### Human Brain Tissue Immunostaining

Frozen postmortem human middle temporal gyrus cortical tissues were obtained from control cases through the Banner Sun Health Research Institute (Table 1) (Wong et al. 2015). The tissues were fixed in 4% PFA overnight at 4 °C, then transferred to 30% sucrose in PBS overnight at 4 °C, sectioned into 40-μm slices, and stored free-floating in cryoprotectant at −20 °C until used for immunohistology. At the time of use, slices were washed with PBS, refixed in 4% PFA for 10 min at 4 °C, washed again in PBS, treated with 1.5% H_2_O_2_ in methanol for 15 min at RT, and washed with PBS. Next, slices were treated with 0.2 M HCl for 8 min at RT followed by 10 μg/mL proteinase K in PBS for 20 min at RT, refixed with 4% PFA for 10 min at 4 °C, and washed with PBS. Slices were then placed into denaturing buffer (50% formamide, 10% dextran sulfate, 1% Denhardt’s Solution, 0.3 M NaCl, 20 mM Tris–HCl pH 8, 5 mM EDTA pH 8, 10 mM NaPO4, and 1% sarcosyl) at 80 °C for 2 min and were incubated on a shaker at 55 °C overnight. The next day, slices were rinsed with 5× saline sodium citrate (SSC) buffer preheated to 65 °C and incubated with 50% formamide in 2× SSC shaking for 30 min at 65 °C. Slices were then washed successively with 2× SSC at 37 °C, 0.1× SSC at RT, and finally PBS at RT and blocked for 1 h at 4 °C in staining buffer (0.05 M Tris pH 7.4, 0.9% NaCl, 0.25% gelatin, 0.5% TritonX-100, and 3% donkey serum). Slices were then incubated with primary antibodies against GFAP (1:250, PhosphoSolutions 621-GFAP), NeuN (1:100, Novus NBP1–92693), AKT1 (1:100, Cell Signaling 2938), AKT2 (1:10, Cell Signaling 2964), or AKT3 (1:100 Cell Signaling 14 982) diluted in staining buffer for 96 h. Following PBS washes, slices were incubated with Hoechst (1:1000) and secondary antibodies conjugated to Alexa Fluor 488, Cy3, or Alexa Fluor 647 (1:250, Jackson ImmunoResearch or Thermo Fisher Scientific) diluted in staining buffer without donkey serum for 3 h at RT. Slices were then washed two times with PBS containing 0.03% TritonX-100 followed by one PBS wash. To block nonspecific lipofuscin autofluorescence, slices were also treated with 0.5% Sudan Black in 70% ethanol for 1 min and washed with PBS prior to being mounted on slides with Mowiol. Z-stacks through the entire thickness of the brain slices were imaged using the Nikon A1R confocal microscope with all microscope parameters held constant across all slices. Images are representative of four independent samples (two subjects per sex) for each staining.

**Table 1:** Patient demographics associated with the human cortical samples used in this study

CTRL	Age	Sex	PMI	Braak stage
1	53	M	3.66	I
2	58	F	3.13	I
3	59	F	3.15	I
4	65	M	3.5	I
Mean	**58.75**		**3.36**	
SD	**4.92**		**0.26**	

Note: CTRL, control subject with no dementia or major neuropathological diagnosis; age (years); M, male; F, female; PMI, postmortem interval (h); Braak stage (I–VI); bold values denote the mean and standard deviation (SD) for age and PMI.

### Western Blotting

Mouse hippocampal tissues were homogenized via sonication in lysis buffer (10 mM HEPES pH 7.4, 150 mM NaCl, 50 mM NaF, 1 mM EDTA, 1 mM EGTA, 10 mM Na_4_P_2_O_7_, 1% Igepal) with 1% protease inhibitor cocktail (Sigma), 1% phosphatase inhibitor cocktails II and III (Sigma), and 1% TritonX-100. Twenty micrograms of protein samples were prepared in Laemmli buffer, separated using 4–12% Bis–Tris gradient gels, and transferred to PVDF membranes. Blots were blocked for 1 h at RT and were incubated with primary antibodies against AKT1 (1:1000, Cell Signaling 2938), AKT2 (1:1000, LSBio LS-C1526232), AKT3 (1:1000, Cell Signaling 8018), and β-tubulin (1:20 000, Abcam ab11308) for up to 72 h at 4 °C in 0.2% I-Block (Tropix) in Tris-buffered saline with 0.1% Tween-20 (TBS-T). Blots were then washed with TBS-T, incubated with HRP-conjugated goat antimouse or antirabbit secondary antibodies (1:5000–1:20 000, Promega) in I-Block solution at RT for 1 h, and washed again with TBS-T. Immunoreactive signals were imaged using enhanced chemiluminescence (GE Healthcare) and normalized by loading control levels. Data are presented as the mean ± SEM relative to the respective WT control levels for n2F3K, c1F3K, and n1F2F mice. WT and mutant data for each double *Akt* isoform mutant line were statistically evaluated by two-tailed Student’s *t*-test using SPSS (IBM Corporation), with *P* < 0.05 considered significant.

## Results

### AKT1 Is Expressed in Interneurons in the Hippocampus and Cortex

**Figure 1:**
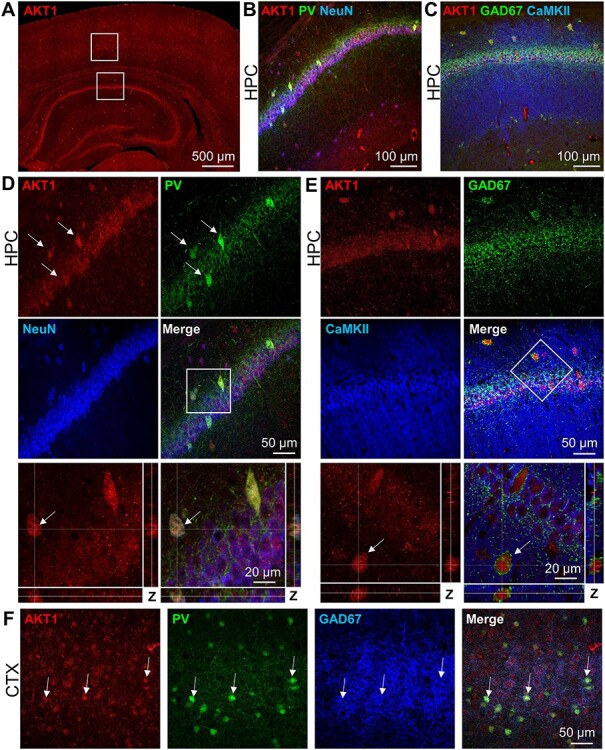
AKT1 is expressed in excitatory neurons and GABAergic interneurons. (*A*) Representative image of AKT1 (red) staining in the hippocampus (HPC) and cortex (CTX) of coronal mouse brain sections. AKT1 shows widespread staining with strong immunoreactivity in the cell body layers of the hippocampus**.** (*B*, *C*) Higher magnification images of area CA1 corresponding to the lower white square in (*A*), showing AKT1 (red) costaining with (*B*) the interneuronal marker PV (green) and neuronal marker neuronal nuclei (NeuN, blue) or (*C*) the interneuronal marker GAD67 (green) and excitatory neuron marker calmodulin-dependent kinase II alpha (CaMKII, blue). (*D*) Higher magnification of SP in (*B*) shows that AKT1 is present in neuronal cell bodies, including PV+ interneurons (arrows). Lower panels: Higher magnification of the white square with profile views of the Z-stack images through a PV+ cell (arrow) confirms that AKT1 colocalizes with PV. (*E*) Higher magnification of SP in (*C*) shows widespread CaMKII staining within the cell body layer as well as the SR and stratum oriens (SO). GAD67 shows perisomatic staining. Lower panels: Higher magnification of the white square with profile views of the Z-stack images through an AKT1+ cell (arrow) in the SO confirms GAD67 staining around the cell body. (*F*) Higher magnification of the somatosensory cortex corresponding to the upper white square in (*A*) confirms that AKT1 (red) staining colocalizes with the GABAergic markers PV (green) and GAD67 (blue). White arrows denote overlapping staining across markers.

Polymorphisms in *AKT1* have been linked to schizophrenia and other complex brain disorders (Emamian et al. 2004; Ikeda et al. 2004; Schwab et al. 2005; Xu et al. 2007). To better understand the role that AKT1 may play in these disorders, we examined its expression pattern in the brain. We recently showed that AKT1 is primarily expressed in the neurons of the mouse hippocampus (Levenga et al. 2017). In that study, we also observed AKT1-positive (AKT1+) cells outside the SP with morphologies consistent with interneurons and located where interneurons are known to be found in the hippocampus. To determine if these AKT1+ cells were interneurons, we costained coronal mouse brain slices for AKT1 and interneuronal cell markers. In agreement with our previous study, we observed robust AKT1 immunostaining in the hippocampus (Fig. 1*A*). Costaining with the GABAergic interneuronal markers PV and GAD67 (Stone et al. 1999; Rudy et al. 2011) showed that neurons in area CA1 positive for PV (Fig. 1*B*) and GAD67 (Fig. 1*C*) had high AKT1 expression. Counterstaining with the neuronal marker NeuN and the excitatory neuronal marker CaMKII showed that AKT1 was also expressed in excitatory neurons (Fig. 1*B*,*C*). Higher magnification of the SP layer confirmed colocalization of AKT1 and PV (Fig. 1*D*). A profile view through the Z-stack of these images showed that AKT1 was found throughout the soma of PV+ cells (Fig. 1*D*). Using the same approach, we also confirmed AKT1 colocalization with the more general interneuronal marker GAD67, which displayed perisomatic staining around the AKT1+ cells (Fig. 1*E*). Counterstaining with CaMKII confirmed that double AKT1+ and GAD67+ cells did not contain the excitatory neuronal cell marker. To determine if AKT1 interneuronal expression extended to other hippocampal regions, we imaged AKT1 and interneuronal markers in the dentate gyrus (DG) and area CA3. Like area CA1, AKT1 colocalized with PV+ and GAD67+ cells in these hippocampal regions (Supplementary Fig. S1*A*). This staining pattern extended to the cortex, where we observed similar patterns of AKT1 signal (Fig. 1*F*). Because we observed some AKT1+ cells with no detectable PV signal, we also stained for SST and CAL to determine if other subpopulations of interneurons (Rudy et al. 2011) expressed AKT1. Like PV, we found AKT1 present within the soma of SST+ cells (Supplementary Fig. S1*B*) and CAL+ cells (Supplementary Fig. S1*C*) in the hippocampus and cortex. These data support the notion that AKT1 is highly expressed in the interneurons of the brain.

### AKT2 Is Expressed in Astrocytes in the Hippocampus and Cortex

**Figure 2:**
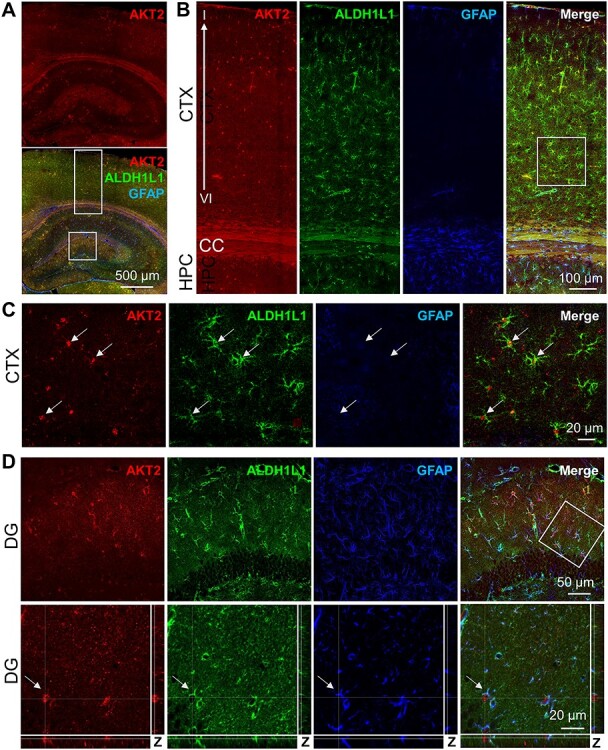
AKT2 is expressed in astroglia. (*A*) Representative image of AKT2 (red) costaining with astrocyte markers ALDH1L1 (green) and glial fibrillary acidic protein (GFAP, blue) in the HPC and CTX of coronal mouse brain sections. AKT2 is present throughout these brain regions, with strong immunoreactivity in the molecular layer of the DG. (*B*) Higher magnification of the rectangular area in (*A*) shows ALDH1L1 expression throughout cortical layers I-VI like AKT2, whereas GFAP is largely absent from the CTX and displays greater immunoreactivity in the HPC. (*C*) Higher magnification of the white square in (*B*) confirms that AKT2 colocalizes with ALDH1L1, while little to no GFAP is detected in the CTX. (*D*) In the DG corresponding to the white square area in (*A*), AKT2 colocalizes with both ALDH1L1 and GFAP and is absent from the neuronal cell bodies in the granule cell layer. Lower panels: Higher magnification of the white square with profile views of the Z-stack images through an AKT2+ cell (arrow) confirms that AKT2 is present in the cell bodies of ALDH1L1+ and GFAP+ astrocytes.

In the brain, AKT2 is implicated in glioblastoma malignancy (Zhang et al. 2009; Wang et al. 2010). We previously showed that AKT2 was expressed in the astrocytes of mouse hippocampal area CA1 using GFAP staining, with AKT2 observed in the astrocyte cell body and GFAP defining astrocytic processes (Levenga et al. 2017). To expand on these findings, we evaluated AKT2 expression in the mouse brain more thoroughly. In agreement with our previous study, staining for AKT2 showed a stronger signal in the molecular layers of DG (Fig. 2*A*). Within the cortex, AKT2 staining was uniformly scattered throughout the layers (Fig. 2*A*,*B*). Specificity of AKT2 signal was verified using slices stained without the primary antibody but with the secondary antibody (Supplementary Fig. S2*A*) as well as using *Akt2* KO tissue (Levenga et al. 2017). To support the idea that AKT2 is expressed in astrocytes, we costained for the astrocyte markers GFAP and ALDH1L1. Although GFAP is an established astrocytic marker, not all astrocytes express detectable GFAP (Hol and Pekny 2015). We, therefore, additionally stained for ALDH1L1, a more general astrocytic marker (Serrano-Pozo et al. 2013). Indeed, we found ALDH1L1 staining uniformly dispersed throughout the cortical layers, but little or no GFAP signal was visible in the cortex (Fig. 2*B*). Despite the low GFAP levels in the cortex, higher magnification images showed that AKT2 colocalized with these ALDHL1+ astrocytes (Fig. 2*C*). By contrast, both ALDH1L1 and GFAP were strongly expressed and overlapped significantly in area CA1, and importantly, AKT2 colocalized with not only GFAP, as we reported previously, but also ALDH1L1 (Supplementary Fig. S2*B*). In the DG similarly, AKT2 colocalized with both ALDH1L1 and GFAP (Fig. 2*D*). Closer examination with the profile views of the Z-stack images confirmed that AKT2 identified the cell body while GFAP and ALDH1L1 mainly stained astrocytic processes (Fig. 2*D*). To assess the specificity of AKT isoform expression in astrocytes, we also performed costaining for ALDH1L1 and GFAP with AKT1. We found no evidence that AKT1 localized with either astrocytic marker in area CA1, indicating that AKT2 but not AKT1 is present in the astrocytes of this brain region (Supplementary Fig. 2*C*). To quantify the cell-type expression differences between AKT1 and AKT2, absolute AKT1 and AKT2 signal intensities in NeuN+ and GFAP+ cells were compared (Supplementary Fig. 3*A*). The results confirmed the expression of AKT1 in neurons and AKT2 in astroglial populations (Supplementary Fig. S3*B*). These data provide further support that astrocytes in the mouse hippocampus specifically expressAKT2.

**Figure 3:**
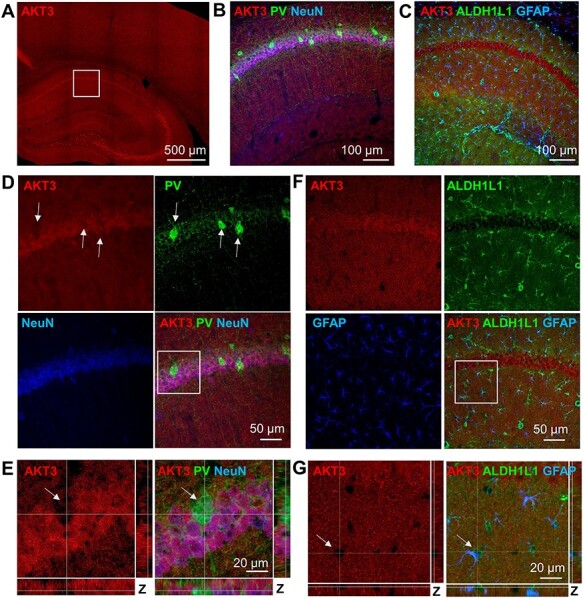
AKT3 is widely expressed in neurons but is not expressed in interneurons or astrocytes of area CA1. (*A*) Representative image of AKT3 (red) staining in the hippocampus and cortex of coronal mouse brain sections. AKT3 is present throughout the brain, showing strong staining in both the cell body and neuropil layers of the hippocampus, especially in area CA3 cell bodies. (*B*, *C*) Higher magnification images of area CA1 corresponding to the white square in (*A*), showing AKT3 (red) costaining with (*B*) the interneuronal marker PV (green) and neuronal marker NeuN (blue) or (*C*) the astrocytic markers ALDH1L1 (green) and GFAP (blue). (*D*) Higher magnification of SP in (*B*) shows that AKT3 staining overlaps with NeuN but not PV+ cells. (*E*) Profile views of the Z-stack images through a PV+ cell (green) in area CA1 SP confirms the absence of AKT3 immunostaining in the interneuron cell body. (*F*) Higher magnification of SP and SR in (*C*) shows that AKT3 does not have a similar staining pattern to ALDH1L1 or GFAP. (*G*) Profile views of the Z-stack images through an ALDH1L1 and GFAP double-positive cell (arrow) in area CA1 SR confirms the absence of AKT3 immunostaining in the astrocyte cellbody.

**Figure 4:**
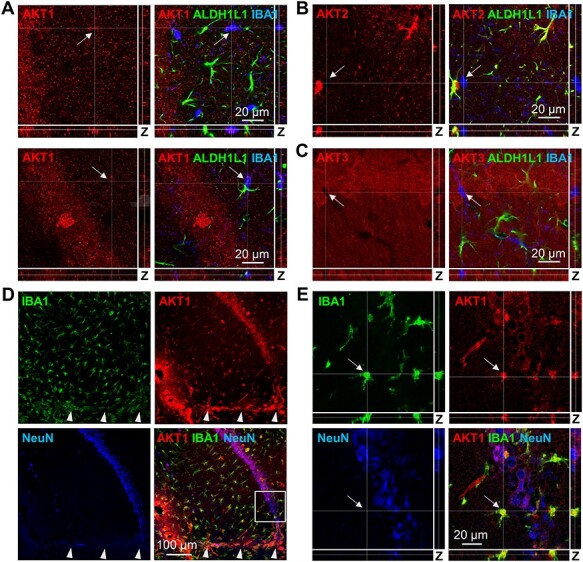
AKT1 is weakly expressed in microglia but increases following brain injury. (*A*) Upper and lower panels: Representative images of AKT1 (red) costaining with the microglial marker IBA1 (blue) and astrocytic marker GFAP (green) in area CA1 at high magnification. Profile views of the Z-stacks through IBA1+ cells show low levels of AKT1 in microglia cell bodies but not astrocytes. (*B*) High magnification image of AKT2 (red) costaining with IBA1 (blue) and ALDH1L1 (green) in area CA1 shows AKT2 colocalization with the astrocytic but not microglial marker. Profile views of the Z-stacks through an IBA1+ cell confirm absence of AKT2 in the microglial cell body. (*C*) High magnification image of AKT3 (red) costaining with IBA1 (blue) and ALDH1L1 (green) in area CA1 shows absence of AKT3 from both microglia and astrocytes. Profile views of the Z-stacks through an IBA1+ cell confirms that AKT3 is not detected in the microglial cell. (*D*) IBA1 (green) staining in hippocampal area CA1 following brain injury shows activation of microglia near the injury site, indicated by their ameboid morphology; arrowheads denote the wound track. Costaining with AKT1 (red) and counterstaining with NeuN (blue) show increased AKT1 signal in microglia compared with uninjured brain tissues (*A*). (*E*) Higher magnification of the white square in (*D*) with profile views of the Z-stack images through an IBA1+ cell (arrow) confirms colocalization with AKT1 immunostaining.

### AKT3 Is Expressed in Overlapping Cell Types with AKT1 but Not in Interneurons or Astrocytes

Mutations in the *AKT3* gene are linked to excessive brain growth called hemimegalencephaly (Alcantara et al. 2017). Furthermore, *Akt3* KO mice display significantly reduced brain and neuron size (Easton et al. 2005). These data suggest that AKT3 is involved in neuronal cell growth. Previously, we found that AKT3 expression overlapped with AKT1 in the neurons of the hippocampus with pronounced immunolabeling in area CA3 (Levenga et al. 2017). To build on this previous work, we further examined the expression pattern of AKT3 in the mouse brain. We observed widespread AKT3 immunostaining throughout the hippocampus and cortex (Fig. 3*A*). Consistent with our previous report, AKT3 was detected in both cellular and neuropil regions of the hippocampus and showed very strong expression in the area CA3 somatic layer (Fig. 3*A*). Quantification of AKT isoform staining intensity in the hippocampus demonstrated both AKT1 and AKT3 signals in the somatic and dendritic layers of CA1 and CA3 (Supplementary Fig. S3*C*). However, the AKT3 signal was strongest in the SP of CA3 compared with the SR and CA1 layers, while AKT1 showed stronger signal in the SP of CA1 (Supplementary Fig. S3*C*). To assess the cell-type localization of AKT3, we costained for the neuronal markers NeuN and PV (Fig. 3*B*) or the astrocytic markers ALDH1L1 and GFAP (Fig. 3*C*). Like AKT1, AKT3 colocalized with NeuN in area CA1 (Fig. 3*D*), indicating that AKT3 was expressed in the neurons. However, unlike AKT1, AKT3 did not colocalize with the GABAergic interneuron marker PV in area CA1, as confirmed by the absence of AKT3 signal from the PV+ cell in the Z-stack profile view (Fig. 3*E*). In contrast to AKT2, we also did not observe AKT3 colocalization with the astrocytic markers ALDH1L1 and GFAP in area CA1 (Fig. 3*F*). Higher magnification imaging of an ALDH1L1+ and GFAP+ cell confirmed the absence of detectable AKT3 in the cell body (Fig. 3*G*). Quantification of absolute AKT3 signal in PV+, NeuN+, and GFAP+ cells confirmed the expression of AKT3 in excitatory neuronal populations (Supplementary Fig. S3*D*). To determine if these observations extended beyond area CA1, we examined costaining for AKT3 and interneuronal or astrocytic markers in the DG, area CA3, and cortex. As with our AKT3 staining results in area CA1, we did not detect AKT3 colocalization with PV and GAD67 (Supplementary Fig. S4*A*) or with ALDH1L1 and GFAP (Supplementary Fig. S4*B*) in the other hippocampal regions. In the cortex, we similarly did not detect AKT3 in the soma of PV+ interneurons (Supplementary Fig. S4*A*) or ALDH1L1+ and GFAP+ astrocytes (Supplementary Fig. S4*B*). These data suggest that AKT3 in the mouse brain is widely expressed in neurons but is limited to excitatory neurons, overlapping with AKT1 in these cells. Whereas AKT1 is also present in interneurons and AKT2 expresses in astrocytes, our data demonstrate that AKT3 is not found in the soma of either of these cell types in the mouse brain.

### AKT1 Is the Only AKT Isoform Detected in Microglia

To determine if microglia expressed one or more AKT isoforms in the mouse brain, we next costained for each isoform with the microglial marker IBA1 and counterstained with ALDH1L1. At lower magnifications, we did not readily observe a coinciding pattern of staining between any AKT isoform and IBA1 (data not shown), so we performed higher magnification imaging of area CA1 and examined the profile view of the Z-stacks. We detected very low levels of AKT1 in microglia using this analysis (Fig. 4*A*). AKT2 colocalized with the astrocytic marker ALDH1L1, as shown earlier (Fig. 2) but was not detectable in IBA1+ cells (Fig. 4*B*). Likewise, although AKT3 showed diffuse staining throughout area CA1, it was absent from IBA1+ cells (Fig. 4*C*). We next tested if AKT1 expression would be more apparent following microglial activation. To do this, we examined the AKT1 signal in IBA1+ cells following brain injury to the hippocampus of sham surgery-treated mice. As expected, IBA1 staining showed microglial activation indicated by a transition of microglia near the injury site to the morphologically distinct ameboid phenotype (Fig. 4*D*), consistent with their known response to injury and inflammation (Wolf et al. 2017). We also observed increased AKT1 signal that colocalized with IBA1+ cells (Fig. 4*D*). Higher magnification imaging near the wound site more clearly demonstrates this overlap (Fig. 4*E*). Therefore, although detection levels were low, these data suggest that microglia in the mouse brain express AKT1 as their primary AKT isoform.

### AKT Isoforms Are Expressed in Similar Cell Types in the Amygdala as the Hippocampus

**Figure 5:**
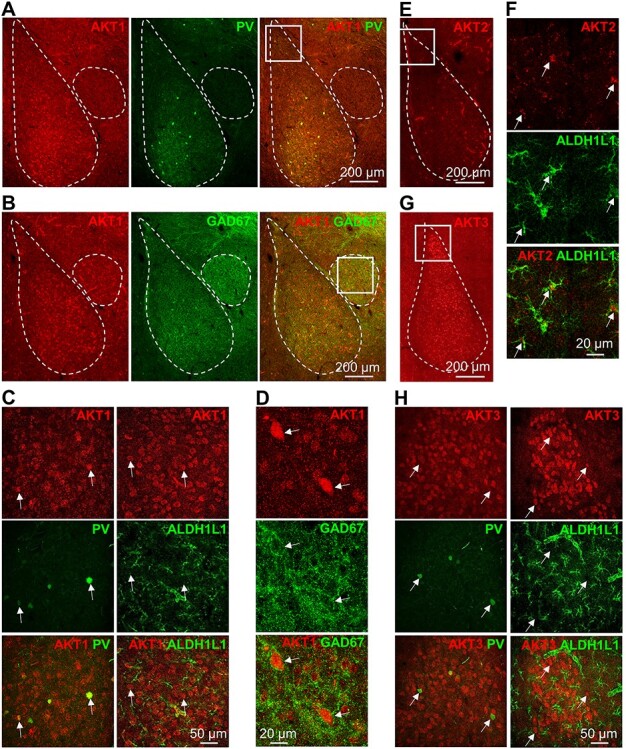
Cell-type expression of AKT isoforms in the amygdala mirrors the expression patterns in hippocampus and cortex. (*A*, *B*) Representative images of the amygdala costained for AKT1 (red) with the interneuronal markers (*A*) PV (green) and (*B*) GAD67 (green). PV is more prominently expressed in the BLA (left outline) compared with the CeA (right outline), but GAD67 staining shows that interneurons are present in both subregions of the amygdala. AKT1 is also present in both subregions. (*C*) Higher magnification images of AKT1 staining in the LA corresponding to the white square in (*A*) shows that PV is detected in cells positive for AKT1 (white arrows, left column), whereas the astrocytic marker ALDH1L1 is not detected (white arrows, right column). (*D*) Higher magnification images of the amygdala corresponding to the white square in (*B*) shows AKT1+ cells (white arrows) with perisomatic GAD67 staining in the CeA. (*E*) Representative image of AKT2 staining in the BLA. (*F*) Higher magnification of the LA corresponding to the white square in (*E*) shows that AKT2 colocalizes with ALDH1L1 staining (white arrows). (*G*) Representative image of AKT3 staining in the BLA. (*H*) Higher magnification of the LA corresponding to the white square in (*G*) shows that PV+ interneurons (white arrows, left column) do not express AKT3. Costaining with ALDH1L1 shows that ALDH1L1 also is not detected in cells positive for AKT3 (white arrows, right column) in theLA.

The amygdala is a critical brain region involved in the manifestation of affective disorders and responses to threats (LeDoux 2007; Sears et al. 2014). Because AKT is involved in the neural responses to therapeutic agents used to treat affective disorders (Beaulieu 2012; Park et al. 2014), we also extended our cell-type expression analysis of AKT isoforms to the amygdala. Because AKT1 colocalized with the interneuronal markers in the mouse hippocampus and cortex (Fig. 1 and Supplementary Fig. S1), we costained the amygdala for AKT1 with PV or GAD67. PV signal was more prominent in the basolateral amygdala (BLA) than the central amygdala (CeA) (Fig. 5*A*). At the same time, GAD67 was present in both the BLA and CeA but with higher expression in the CeA (Fig. 5*B*), consistent with the high concentration of interneurons in this brain region (Sosulina et al. 2010). Higher magnification imaging of the lateral amygdala (LA) showed AKT1 immunoreactivity in PV+ cells (Fig. 5*C*), as found in the hippocampus and cortex. Like these brain regions, AKT1 also was observed in the neuronal cell bodies of the LA, indicated by colocalization with NeuN (data not shown) but not with the astrocytic marker ALDH1L1 (Fig. 5*C*). In the CeA, AKT1 was observed in neuronal cell bodies with perisomatic GAD67 signal (Fig. 5*D*), as found in the hippocampus (Fig. 1*E*). These data support the idea that AKT1 is expressed in the GABAergic interneurons and other neuronal populations but not in the astrocytes of the mouse brain. To examine whether AKT2 was expressed in the astrocytes of the amygdala, we stained for AKT2 and imaged the LA (Fig. 5*E*). Costaining with ALDH1L1 showed that AKT2 colocalized with ALDH1L1+ cells in the LA (Fig. 5*F*). We also stained for AKT3 in the amygdala (Fig. 5*G*) and observed that AKT3 colocalized with NeuN (data not shown), like AKT1, but not with PV or ALDH1L1 in the LA (Fig. 5*H*). These data suggest that AKT1 and AKT3 expression overlap in excitatory neurons, but AKT1 is selectively expressed in interneurons while AKT2 is the only isoform expressed in astrocytes of the mouse amygdala. Thus, AKT isoform expression in the amygdala is directed in a pattern like other brain regions.

### Human Brain Tissue Displays Cell-Type-Specific AKT Isoform Expression

**Figure 6:**
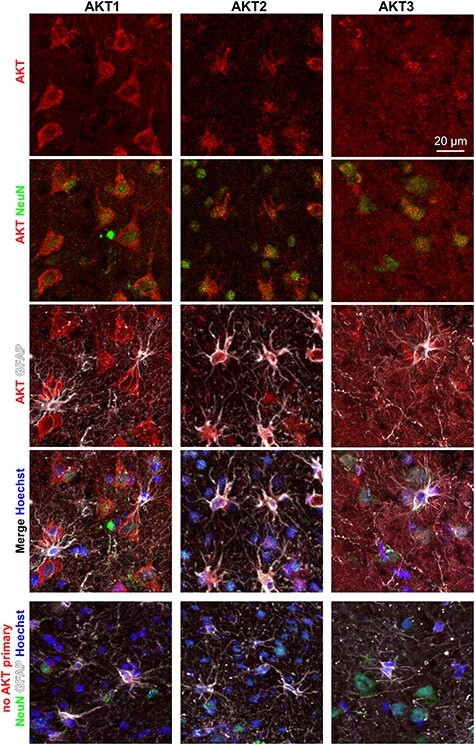
AKT isoform expression in human brain tissue. Immunofluorescent staining using isoform-specific AKT antibodies and cell-type-specific markers in human brain tissue isolated from the temporal cortex shows an expression pattern like our mouse brain staining. AKT1 (red, left column) shows colocalization with the neuronal marker NeuN (green), AKT2 (red, middle column) shows colocalization with the astrocytic marker GFAP (white), and AKT3 (red, right column) shows diffuse staining in neuronal cell bodies and neuropil throughout the tissue. Bottom row: Human cortical tissue costained for NeuN (green) and GFAP (white) with AKT isoform-specific primary antibodies omitted but the secondary antibody included (red) to demonstrate the specificity of AKT isoform staining. Hoechst (blue), nuclear stain. Specimens were obtained from four subjects (*N* = 2/sex) considered as controls by the brain bank with a mean age of 58.75 years (Table 1), and images are representative of AKT isoform staining patterns for each subject.

To determine if cell-type specificity of the AKT isoform expression in the mouse brain was also observed in the human brain, we immunostained brain tissue sections prepared from human temporal cortex for the different AKT isoforms in the context of neural cell markers. All three AKT isoforms were detected in the human brain tissue (Fig. 6). AKT1 identified the soma of cells with recognizable neuronal cell body morphology. AKT2 also identified cell bodies, but these cells had notably different morphology than AKT1+ cells. AKT3 identified some cell bodies but had a much more diffuse staining pattern, like what we observed in the mouse hippocampus’s neuropil layers. In agreement with our mouse brain results, we found that AKT1 colocalized with the neuronal marker NeuN but not the astroglial marker GFAP in human brain tissue (Fig. 6). Because PV+ interneurons selectively expressed the AKT1 isoform in mice (Fig. 1), we next costained for AKT1 and PV in the human cortex. In agreement with our mouse observations, we found AKT1 in PV+ neurons (Supplementary Fig. S5*A*). AKT2, inversely, colocalized with GFAP but not NeuN (Fig. 6), as observed in the mouse brain. AKT3 showed somatic staining that colocalized with NeuN+ but not GFAP+ cell bodies in the human brain tissue (Fig. 6), which is in agreement with our mouse brain data. One possible explanation for the diffuse staining pattern of AKT3 is that it is more widely distributed throughout the neuron. To test this idea, we next costained for microtubule-associated protein 2 (MAP2), a neuron-specific cytoskeletal protein enriched in dendrites. Consistent with the notion that AKT3 is also distributed in dendritic processes, we found colocalization of AKT3 with MAP2 immunoreactivity (Supplementary Fig. S5*B*). Similar expression patterns for AKT isoforms between the mouse and human brain tissue support mouse models for translational work related to AKT function.

### AKT Compensation in Response to Genetic Deletion of AKT Isoforms

**Figure 7:**
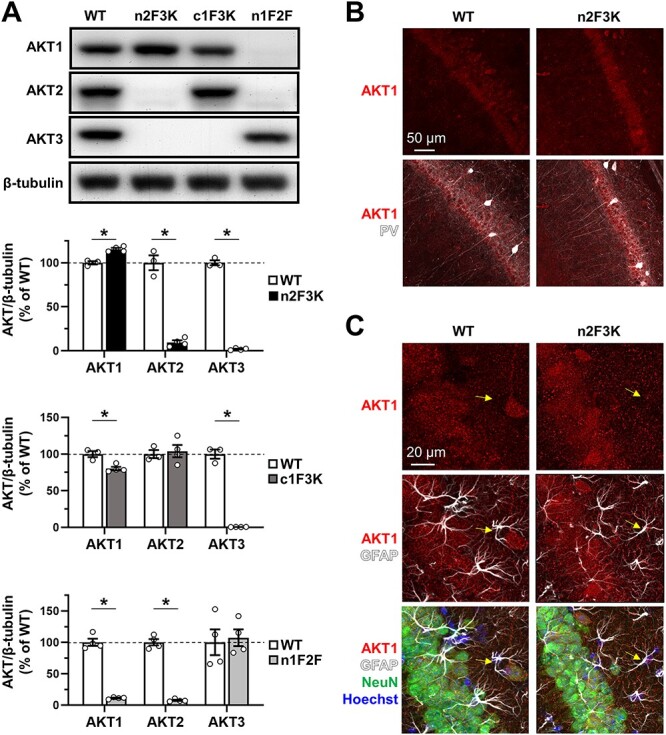
AKT isoform expression in response to genetic deletion of the other two isoforms. (*A*) Western blot analysis of AKT isoform levels in hippocampal lysates prepared from *Akt* mutant mice. Cre-mediated removal of floxed *Akt2* under the early neural progenitor *Nes* promoter in an *Akt3* KO background (n2F3K) led to near-complete removal of both AKT2 and AKT3 in the hippocampus (AKT2: *t*(5) = 12.07, *P* < 0.001; AKT3: *t*(5) = 40.15, *P* < 0.001). This genetic manipulation also led to a significant increase in the AKT1 levels in n2F3K mice when compared with WT controls (*t*(5) = 6.471, *P* = 0.001). Cre-mediated removal of floxed *Akt1* under the excitatory neuron *Cam2α* promoter in an *Akt3* KO background (c1F3K) led to a small reduction in AKT1 levels in the hippocampus (*t*(5) = 4.310, *P* = 0.008) with complete AKT3 loss (*t*(5) = 16.99, *P* < 0.001) but no change in AKT2 levels (*t*(5) = 0.340, *P* > 0.05). *Nes*-Cre-mediated removal of floxed *Akt1* and *Akt2* (n1F2F) resulted in near-total removal of AKT1 (*t*(6) = 15.45, *P* < 0.001) and AKT2 (*t*(6) = 17.80, *P* < 0.001) but no significant change in AKT3 expression (*t*(6) = 0.297, *P* > 0.05). *N* = 3–4/genotype. (*B*) AKT1 staining in area CA1 of n2F3K mice shows a similar pattern to WT controls except AKT1 immunoreactivity is increased in cell bodies, including that of PV+ interneurons, within the n2F3K hippocampus. AKT1 (red), PV (white). (*C*) Higher magnification of AKT1 costaining with the astrocytic marker GFAP (white) in area CA1 shows that AKT1 expression is not detected in astrocytes (yellow arrows) of both n2F3K mice and WT controls. Hoechst (blue), nuclear stain. ^*^P < 0.05.

Our data show cell-type-specific expression patterns of AKT isoforms under normal conditions. We next examined whether these patterns were responsive to changes in the other two AKT isoforms’ expression. In this way, we could determine if, for example, astrocytes could express another AKT isoform under conditions of AKT2 deficiency. We employed genetic KO and Cre-mediated deletion of *Akt* isoforms to remove two AKT isoforms at a time from the mouse brain. We generated three double *Akt* isoform mutants in this manner and performed western blot analysis of hippocampal tissue from these mice to measure each isoform’s relative expression. With the early neural progenitor Cre driver *Nes*-Cre to delete *Akt2* in an *Akt3* KO background (n2F3K), both AKT2 and AKT3 levels were undetectable in the hippocampus (Fig. 7*A*). Interestingly, AKT1 was significantly increased in n2F3K mice compared with WT controls (Fig. 7*A*). We next used Cre-mediated *Akt1* deletion under the *Camk2a* promoter to remove AKT1 selectively from excitatory neurons in an *Akt3* KO background (c1F3K). In c1F3K mice, we did not detect AKT3 and found modest reductions in the AKT1 levels in the hippocampus (Fig. 7*A*). AKT2 levels were not affected in these mice (Fig. 7*A*). Finally, with *Akt1* and *Akt2* removal under *Nes-*Cre (n1F2F), we observed near-total removal of AKT1 and AKT2 but no significant difference in the AKT3 levels when compared with controls (Fig. 7*A*). These results suggest that AKT1 levels increase to compensate for the loss of AKT2 and AKT3 in the hippocampus.

To examine the increased AKT1 levels’ cellular localization, we costained brain sections from the n2F3K mice for AKT1 and cell-type-specific markers. AKT1 showed stronger overall immunoreactivity in the hippocampus of n2F3K mice than WT controls (Fig. 7*B*), which is consistent with our western results. PV staining showed that AKT1 expression in the n2F3K hippocampus overlapped with PV+ interneurons in a manner indistinguishable from controls (Fig. 7*B*). Higher magnification imaging of AKT1 staining with GFAP showed that AKT1 was not detectable in astrocytes, while AKT1 showed colocalization with the neuronal marker NeuN (Fig. 7*C*). These results indicate that AKT1 expression in the n2F3K hippocampus was maintained in the same cell types as WT controls. We also found no difference in the number of PV+ interneurons in area CA1 between n2F3K and WT mice (data not shown), suggesting that the increased AKT1 levels in n2F3K mice were not due to increased numbers of AKT1+ interneurons. Taken together, our findings indicate that the increase in AKT1 levels occurred in the same cell types that generally express AKT1. These data support the idea that under conditions of AKT2 and AKT3 deficiency, AKT1 compensation occurs to a limited extent but is limited to cell types in which AKT1 is typically expressed. These data also suggest that cell-specific expression of AKT isoforms is maintained under different genetic contexts.

## Discussion

These studies provide the first data supporting cell-specific AKT isoform expression in the brains of both fully developed mice and humans. They establish that AKT1 is expressed in excitatory neurons and is selectively expressed in some interneuronal populations in the mouse hippocampus and cortex. Interestingly, when AKT1 was selectively removed from excitatory neurons, total AKT1 levels were only modestly reduced (Fig. 7). This finding, combined with the observation that AKT1 is highly expressed in PV+ interneurons, suggests a critical role for this isoform in GABAergic interneurons. Our data also show that AKT1 is the only isoform detected in microglia, but its expression is very low when compared with other neural cell types in which AKT1 is expressed under normal conditions. Following brain injury, AKT1 levels in microglia increase, perhaps indicating that AKT1 in these cell types is regulated by immune activation. AKT2 is only expressed in astrocytes and is the sole AKT isoform detected in this brain cell type. AKT3 expression overlaps with AKT1 in excitatory neurons but, unlike AKT1, is not seen in interneurons or microglia. AKT3 also differs from AKT1 in that it appears to have a greater expression in the hippocampus’s dendritic layers when compared with AKT1. Additionally, AKT3 is detected at higher levels in the cell bodies of area CA3 than area CA1, a pattern opposite to AKT1. Importantly, we translated our mouse observations to humans by providing evidence of similar cell-type-specific expression patterns of AKT isoforms in the human adult cerebral cortex. Finally, using genetic approaches, we provide evidence that the molecular barriers restricting cell-type expression and localization of AKT isoforms remain intact even in the context of multiple *Akt* isoform deficiencies.

### AKT and Disease

AKT is a critical signaling factor in several neurological diseases and disorders and plays a role in transducing neural signaling in response to a therapeutic treatment. Individual isoforms are associated with different pathological conditions. *AKT1* genetics and function are implicated in schizophrenia, affective disorders, and autism (Emamian et al. 2004; Ikeda et al. 2004; Schwab et al. 2005; Xu et al. 2007; Tan et al. 2008; Karege et al. 2010; Sharma et al. 2010; Blasi et al. 2011; Karege et al. 2012; Ellsworth et al. 2013; Pereira et al. 2014; Schizophrenia Working Group of the Psychiatric Genomics 2014). Additionally, rare *AKT1* variants contribute to metastasis in the brain (Kircher et al. 2019). *AKT2* is linked to glioblastomas, brain glucose metabolism, glial migration, protective responses to ischemia, and the BBB’s maintenance (Lee et al. 2012; Latva-Rasku et al. 2018; Beard Jr et al. 2020; Miao et al. 2020). AKT3 activity also affects brain growth (Easton et al. 2005; Poduri et al. 2012; Dobyns and Mirzaa 2019) and mediates neuroinflammatory responses following injury (Tsiperson et al. 2013; DuBois et al. 2019). Genome-wide association studies (GWAS) link single nucleotide polymorphisms (SNPs) in *AKT3* to schizophrenia (Schizophrenia Working Group of the Psychiatric Genomics 2014). *AKT3*, like *AKT2*, is also linked to glioblastomas (Mure et al. 2010; Xia et al. 2019). Significantly, AKT activity is involved in signaling responses to pharmacological agents used for treatment. Haloperidol, lithium, and clozapine activate AKT in humans or are known to be required for therapeutic responses in animal models (Beaulieu et al. 2004, 2009; Emamian et al. 2004; Alimohamad et al. 2005; Li et al. 2007). Antidepressants are also known to activate AKT signaling (Park et al. 2014), and AKT signaling is abnormal in the brains of postmortem suicide victims (Dwivedi et al. 2010). Furthermore, AKT signaling is critical to neuroprotective activity following stroke (Zhang et al. 2007; Xie et al. 2013). Recently, AKT isoform-specific inhibitors were characterized (Yap et al. 2011; Tei et al. 2015), enabling more studies to differentiate the AKT isoform activities. Improved understanding of cell-type expression patterns for AKT isoforms may allow for even more precise therapeutic targeting of AKT signaling in disease by enabling the development of cell-specific delivery methods, identification of cell-specific AKT-dependent signaling, or use of parallel treatment approaches targeting cell types to gain synergistic benefits.

### AKT1

AKT1 is the most critical of the three AKT isoforms; mice can survive with a single copy of AKT1 in the absence of the other isoforms (Dummler et al. 2006). *Akt1* KO mice have growth retardation and increased neonatal death (Easton et al. 2005; Yang et al. 2005). Supporting that importance, our data suggest that AKT1 is the most diversely expressed isoform in the brain and the sole detectable AKT isoform in both interneurons and microglia (Figs 1 and 4 and Supplementary Fig. S1). SST+ and PV+ cells comprise the largest two populations of GABAergic interneurons, critical to the maintenance of excitatory/inhibitory (E/I) balance in the brain (Zhou et al. 2009; Xue et al. 2014). E/I imbalance has been implicated in many neuropsychiatric and neurodevelopmental disorders (Selten et al. 2018; Sohal and Rubenstein 2019). The selective expression of AKT1 in interneurons suggests an essential role for this isoform in interneuronal function and, therefore, in neurological disorders with E/I imbalances. In support of this idea, *Akt1* KO mice exhibit impaired late-phase long-term potentiation and reduced responsiveness to convulsants that act through interneurons (Chang et al. 2016; Levenga et al. 2017; Wong et al. 2020). Given the broad expression of AKT1 in neural cell types, AKT1 might be expected to exert the most significant effects on cognition and behavior. Supporting this idea, *Akt1* KO mice display alterations in affective behavior, spatial memory, and fear extinction (Wong et al. 2020). AKT1 has received a lot of scientific attention in schizophrenia with early human genetic and mouse studies identifying links between the disorder and AKT1 (Ikeda et al. 2004; Tan et al. 2008; Blasi et al. 2011; Karege et al. 2012). More recent larger-scale genetic studies have failed to link AKT1 to schizophrenia (Loh et al. 2013; Purcell et al. 2014; Farrell et al. 2015; Johnson et al. 2017). However, AKT1 activity has been consistently associated with neural responses to therapies for schizophrenia and other neurological disorders (Chalecka-Franaszek and Chuang 1999; De Sarno et al. 2002; Beaulieu et al. 2004; Emamian et al. 2004; Alimohamad et al. 2005; Li et al. 2007; Beaulieu et al. 2008; Beaulieu et al. 2009; Nciri et al. 2013). Our expression data support the idea that AKT1 may exert these effects through modulating multiple cell-type functions. Future studies aimed at targeting AKT1 in one cell type may help clarify the specific effects of AKT1 activity.

### AKT2

*Akt2* KO mice suffer from a type 2 diabetes-like syndrome (Cho et al. 2001; Garofalo et al. 2003) and display some behavioral abnormalities (Leibrock et al. 2013). However, studies linking glioblastoma to AKT2 provide a valuable clue to the cell type in which this isoform is functionally important (Zhang et al. 2009; Wang et al. 2010). This idea is supported by transcriptomic studies finding *Akt2* as the sole AKT isoform in astroglia (Zeisel et al. 2015). Our new immunostaining data provide further evidence that AKT2 is likely the sole AKT isoform expressed in astroglia of the developed brain (Figs 2 and 5). Additionally, in all mouse brain areas we examined, neurons did not exhibit AKT2 immunolabeling. Consistent with this finding, we showed previously that conditional *Akt2* removal from excitatory neurons failed to reduce AKT2 levels, but removal from neural progenitor cells completely abolished AKT2 expression in the mouse hippocampus (Levenga et al. 2017). These data strongly suggest that AKT2 is specifically expressed in astrocytes, and it is a feature throughout the brain (Figs 2, 5, and 6). Although we observed astrocyte-specific AKT2 expression in both fully developed mouse and human brains, AKT2 expression in human nervous system tissues is not well understood. Different expression patterns are reported in the embryonic, cell culture, and genomic studies (Zeisel et al. 2015). Additional work examining AKT2 expression in the human brain tissue from different developmental stages will be necessary. Our results also suggest that AKT2 expression is highest in astrocytes that express high levels of GFAP (Fig. 2), supporting the idea that AKT2 is found in metabolically active astrocytes. This opens the possibility that AKT2 expression is also regulated by astrocytic activation and may be differentially influenced by pathological states. Future studies examining AKT2 expression following neurological injury or disease may help to resolve this question.

### AKT3

AKT3 is broadly expressed in the body but shows the highest protein expression in the brain (Yang et al. 2005), and *Akt3* KO mice show decreased brain size (Easton et al. 2005; Tschopp et al. 2005). AKT3 is likely crucial for cognition and behavior, as GWAS studies have identified AKT3 in the manifestation of schizophrenia (Schizophrenia Working Group of the Psychiatric Genomics 2014). Interestingly, however, *Akt3* KO mice are reported to have mild or no behavioral phenotypes (Bergeron et al. 2017; Howell et al. 2017; Wang et al. 2017), and we demonstrated previously that *Akt3* KO mice have normal synaptic plasticity in the hippocampus (Levenga et al. 2017; Wong et al. 2020). We also showed that AKT3 expression overlaps with AKT1 in the neurons of the hippocampus (Levenga et al. 2017), suggesting redundant functions. Indeed, we found that AKT1 can compensate for the loss of AKT3 activity to mediate normal expression of metabotropic glutamate receptor-dependent long-term depression in the hippocampus (Levenga et al. 2017) and normal memory (Wong et al. 2020). However, we also found evidence for differential localizations of AKT3 and AKT1 within hippocampal neurons and subregions (Levenga et al. 2017), implying isoform-specific functions. In support of this idea, neural activity-dependent translation requires AKT1 but not AKT3 (Levenga et al. 2017). Also supporting this idea, we now report that AKT3 and AKT1 expression overlaps in excitatory neurons, but unlike AKT1, AKT3 is absent from the interneuronal cell bodies in the mouse brain (Figs 3 and 5, and Supplementary Fig. S4). Another report recently demonstrated that AKT3 plays a critical role in experimental autoimmune encephalomyelitis (EAE) and that AKT3 but not AKT1 expression in oligodendrocytes was essential for the manifestation of EAE (DuBois et al. 2019). Microglia are also important mediators of inflammation in the brain (Barres 2008; von Bernhardi et al. 2016), although they have other vital roles unrelated to immune function (Salter and Beggs 2014). Interestingly, we only detected very low levels of AKT1 in microglia (Fig. 4*A*), but AKT1 levels increased following microglial activation in response to brain injury (Fig. 4*E*). This observation suggests that AKT isoform expression may be activity-regulated. Future efforts to examine if different neural stimuli, such as immunological challenge, initiation of inflammation, or memory formation, alter AKT isoform expression patterns may address this question.

## Conclusion

While it is well known that the three different AKT isoforms are expressed in the brain, the functional significance for the three isoforms is poorly understood. Our results identify that the different AKT isoforms show remarkable neural cell-type expression specificity. These results provide the basis for more extensive and detailed future studies that will further define the importance of each AKT isoform in normal and pathological brain activities. With the availability of isoform- or cell-type-specific interventions and therapeutics, elucidating cell-type-specific AKT isoform activities may allow for improved and more specific therapies that target AKT signaling.

## Supplementary Material

Akt_immunostaining_paper_Supplemental_Material_for_Review_Final_4_19_21_tgab036

## Notes

The human brain tissues were provided by the Brain and Body Donation Program at Banner Sun Health Research Institute in Sun City, AZ. We thank Andrew Cooper-Sansone, Bailey Keller, and Stephanie Quintana for technical assistance with this work. *Conflict of Interest*: None declared.
