# Supplementary material for: Immunohistological Examination of AKT Isoforms in the Brain: Cell-Type Specificity That May Underlie AKT’s Role in Complex Brain Disorders and Neurological Disease
Source: Cereb Cortex Commun. 2021 May 28;2(2):tgab036. doi: 10.1093/texcom/tgab036 (PMC8223503; doi:10.1093/texcom/tgab036)
Supplement: Akt_immunostaining_paper_Supplemental_Material_for_Review_Final_4_19_21_tgab036 [file akt_immunostaining_paper_supplemental_material_for_review_final_4_19_21_tgab036.zip › Akt_immunostaining_paper_Supplemental_Material_for_Review_Final_4_19_21_tgab036.docx]

**
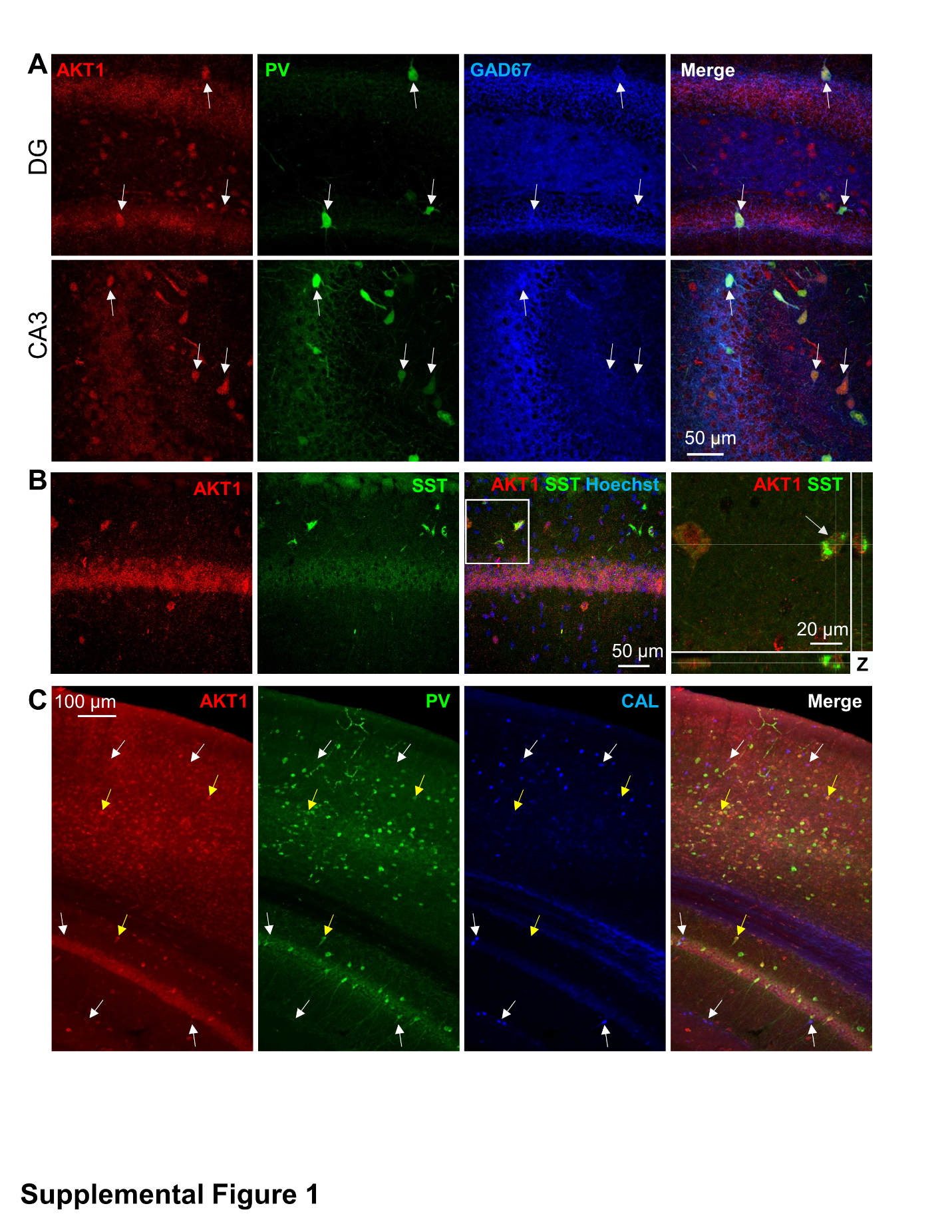
**

**Supplemental Figure 1. AKT1 is expressed in GABAergic interneurons of the hippocampus and cortex. (A)** AKT1 (red) staining colocalizes with the GABAergic markers PV (green) and GAD67 (blue) in the DG and area CA3 of the hippocampus. White arrows denote overlapping staining across markers. **(B)** AKT1 (red) co-staining with the interneuronal marker somatostatin (SST, green) shows that AKT1 is also present in the SST-expressing subclass of interneurons in area CA1. Hoechst (blue), nuclear stain. *Right panel*, Higher magnification of the white square confirms overlap of SST and AKT1 staining in the profile view of the z-stack images through the soma of an SST+ cell (arrow). **(C)** Representative image of calretinin (CAL, blue) staining, which identifies another subclass of GABAergic interneurons, in hippocampal area CA1 and the cortex, shows that AKT1 (red) is also present in CAL+ cells. Co-staining for PV (green) staining confirms that CAL+ cells (white arrows) do not overlap with PV+ cells (yellow arrows) but both interneuronal populations show colocalization with AKT1.

**
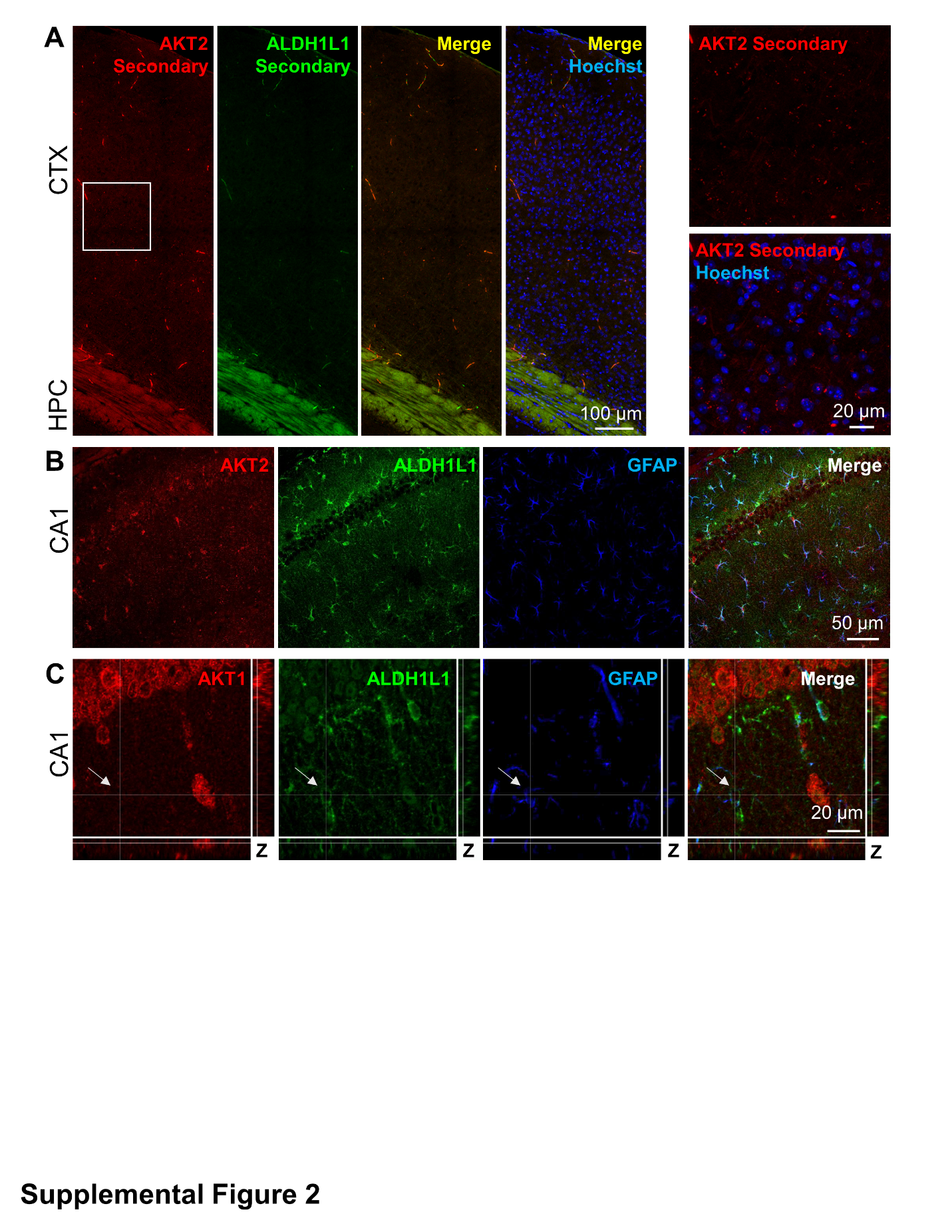
**

**Supplemental Figure 2. AKT2 but not AKT1 colocalizes with astroglial markers in area CA1. (A)** Representative image of background staining in the mouse cortex (CTX) and hippocampus (HPC) when the AKT2 and ALDH1L1 primary antibodies are omitted but the respective secondaries are included. *Right column*, Higher magnification of the white square. Hoechst (blue), nuclear stain. **(B)** AKT2 (red) colocalizes with the astroglial markers ALDH1L1 (green) and GFAP (blue) throughout CA1 and is absent from the neuronal cell bodies in SP. **(C)** High magnification of area CA1 shows that AKT1 does not colocalize with ALDH1L1 and GFAP. Profile views of the z-stack images through an astrocyte identified with ALDH1L1 and GFAP immunostaining (white arrow) confirms that AKT1 is not present in the cell.

**
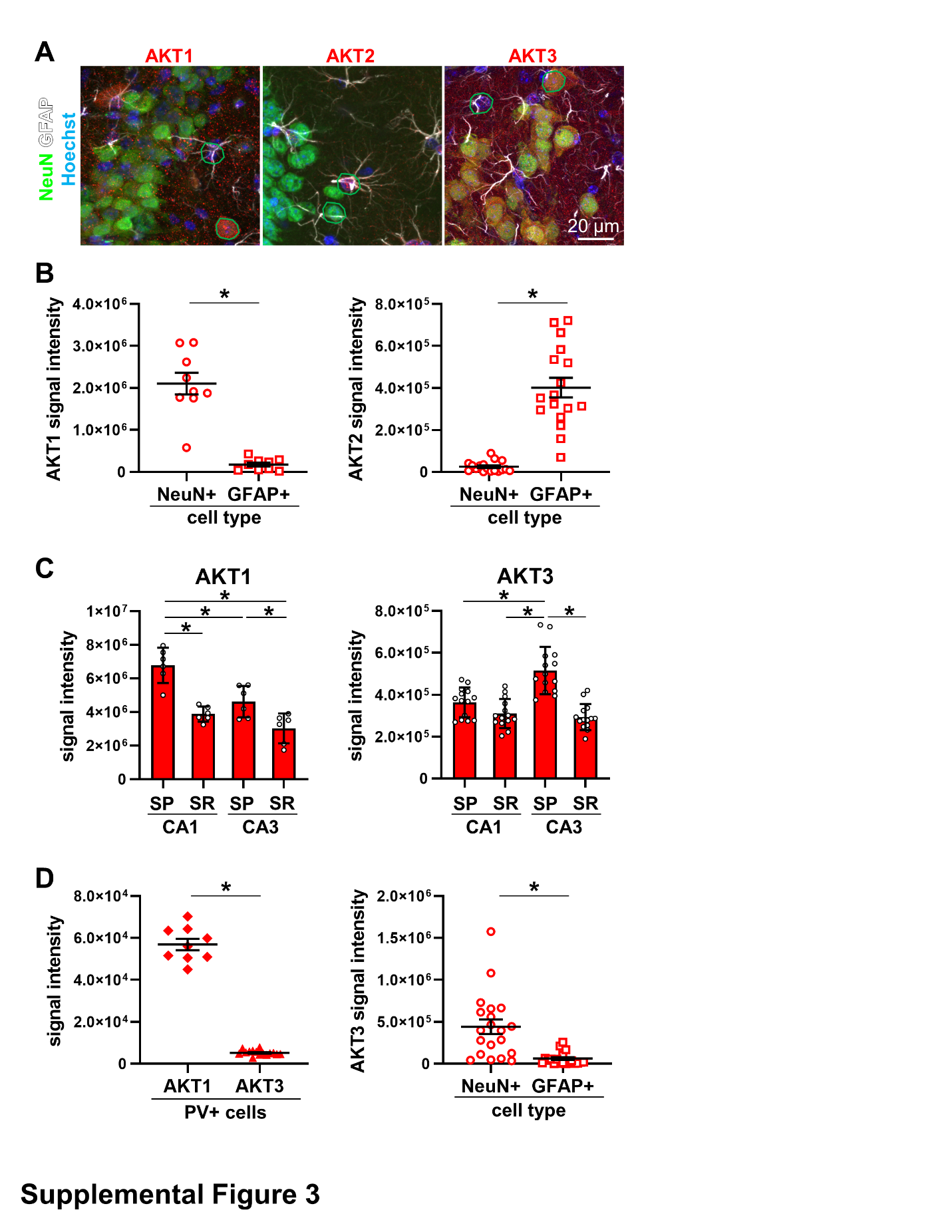
**

**Supplemental Figure 3. Analysis of AKT isoform signal intensity in different cell types and subregions of the hippocampus. (A)** Representative images showing regions of interest (ROI, green outlines) used for measuring AKT isoform signal (red) in different cell types. ROIs were created enclosing the soma of neurons identified by NeuN (green), astrocytes identified by GFAP (white), or interneurons identified by PV (not shown) staining and colocalized with Hoechst-labeled nuclei (blue). **(B)** Comparison of AKT1 (*left*) and AKT2 (*right*) signal in NeuN+ versus GFAP+ cells. AKT1 signal is greater in NeuN+ cells (t(16)=7.334, p<.001) while AKT2 signal is greater in GFAP+ cells (t(32)=7.997, p<.001). N= 3-4 mice, 3-5 cells of each type per mouse. **(C)** Comparison of AKT1 and AKT3 signal in a 2000-μm^2^ ROI in the stratum pyramidale (SP) or stratum radiatum (SR) of area CA1 and CA3. AKT1 signal is greater in the SP than SR, with the strongest signal in CA1 SP (F(3,20)=20.817, p<.001; post hoc comparisons: CA1 SP vs. SR p<.001, CA1 SP vs. CA3 SP p=.002, CA1 SP vs. CA3 SR p<.001, CA3 SP vs. SR p=.022) whereas AKT3 signal is strongest in CA3 SP (F(3,52)=21.094, p<.001; post hoc comparisons: CA3 SP vs. CA1 SP p<.001, CA3 SP vs. CA1 SR p<.001, CA3 SP vs. CA3 SR p<.001). N= 3 mice, 2-4 hippocampal slices per mouse. **(D)** Quantification of AKT3 signal in different cell types. *Left graph*, AKT1 is enriched in PV+ cells compared with AKT3 (t(22)=24.44, p<.0001). *Right graph*, AKT3 signal is greater in NeuN+ than GFAP+ cells (t(38)=4.278, p=.0001). N= 3-4 mice, 3-5 cells of each type per mouse. *p<.05.

**
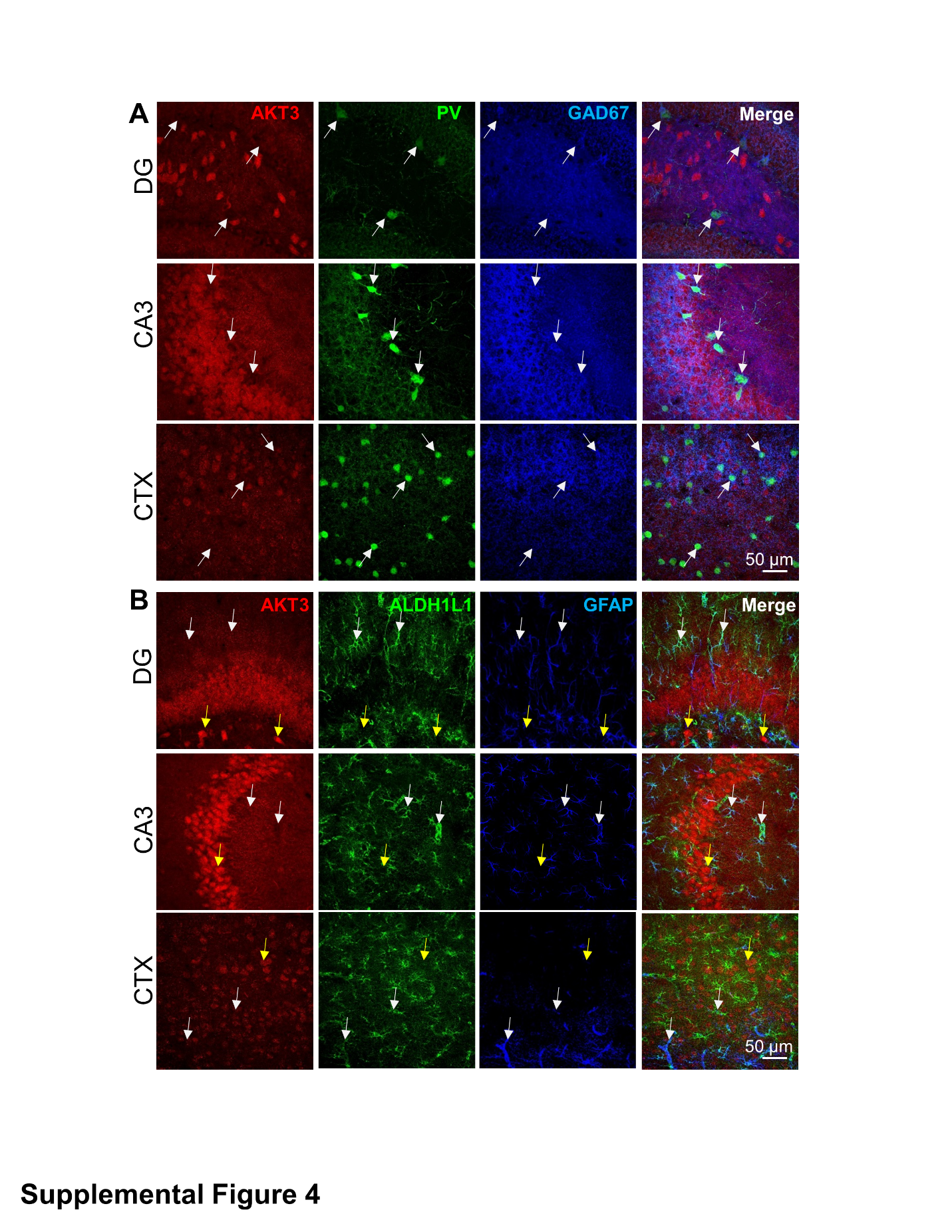
**

**Supplemental Figure 4. AKT3 is expressed in other hippocampal subregions and the cortex similarly to hippocampal area CA1. (A)** AKT3 (red) staining does not colocalize with the GABAergic markers PV (green) and GAD67 (blue) in the DG and area CA3 of the hippocampus or in the somatosensory cortex (CTX). White arrows denote PV and GAD67 double-positive interneurons. These cells show no overlap with AKT3. **(B)** AKT3 (red) staining also does not colocalize with the astrocytic markers ALDH1L1 (green) and GFAP (blue). White arrows denote ALDH1L1 and GFAP double-positive astrocytes. These cells show no overlap with AKT3. Yellow arrows denote AKT3+ cells. These cells show no overlap with ALDH1L1+ and GFAP+ astrocytes.

**
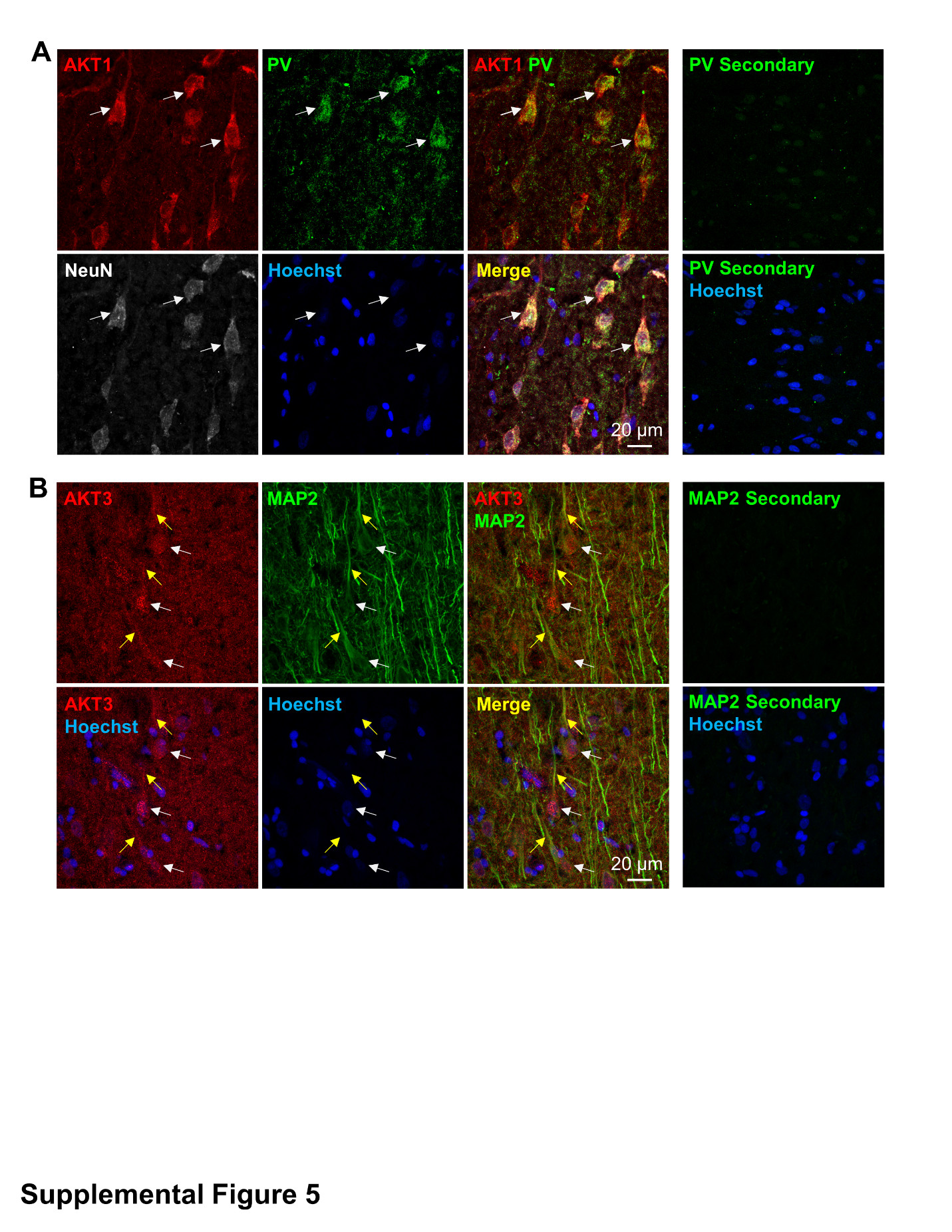
**

**Supplemental Figure 5. Cellular localization of AKT1 and AKT3 in the human brain.** **(A)** AKT1 is expressed in interneurons in the human brain. Co-staining for AKT1 (red) with the interneuronal marker PV (green) and neuronal marker NeuN (white) in human brain tissue from the temporal cortex shows AKT1 colocalization with PV. White arrows denote overlapping staining across markers. Hoechst (blue), nuclear stain. *Right column*, Representative image of human cortical tissue treated with only secondary antibodies to demonstrate the specificity of PV signal. Background staining in the PV channel (green) is shown. **(B)** AKT3 is found in dendritic processes in the human brain. Co-staining for AKT3 (red) with the dendritic marker MAP2 (green) shows that AKT3 colocalizes with both neuronal soma (white arrows) and dendritic processes (yellow arrows). Hoechst (blue), nuclear stain. *Right column*, Representative image of human cortical tissue treated with only secondary antibodies to demonstrate the specificity of MAP2 signal. Background staining in the MAP2 channel (green) is shown.
